# Influence of socioeconomic status on access to temporal artery biopsy and rates of biopsy positivity in patients with suspected giant cell arteritis

**DOI:** 10.1186/s41927-025-00503-0

**Published:** 2025-05-14

**Authors:** Suellen Anne Lyne, Susan Lester, Oscar Kenneth Russell, Carlee Deanne Ruediger, Kathryn Dyer, Jem Ninan, Ernst Michael Shanahan, Catherine Louise Hill

**Affiliations:** 1 School of Medicine, The University of Adelaide, North Terrace, Adelaide, 5000 South Australia; 2 The Queen Elizabeth Hospital, 28 Woodville Road, Woodville South, Adelaide, 5011 South Australia; 3 Flinders Medical Centre, Flinders Drive, Bedford Park, 5042 South Australia; 4 The Lyell McEwin Hospital, Haydown Road, Elizabeth Vale, 5112 South Australia; 5 The Royal Adelaide Hospital, Port Road, Adelaide, 5000 South Australia

**Keywords:** Giant cell arteritis, Temporal arteritis, Vasculitis, Health status, Risk factors, Employment

## Abstract

**Background:**

Data regarding the relationship between socioeconomic status (SES) and incidence of Giant Cell Arteritis (GCA) is conflicting. No previous studies have explored whether SES influences the likelihood of undergoing temporal artery biopsy (TAB). The aim of this study was to determine whether SES influences access to TAB and rate of biopsy positivity in those with suspected GCA.

**Methods:**

This retrospective study included consecutive patients who underwent TAB examined at SA Pathology between 2017 and 2022; age ≥ 50 years and resident in South Australia (SA). Patients’ addresses were used to identify precise geographical areas. Area-level SES was determined using Index of Relative Socioeconomic Advantage and Disadvantage (IRSAD) scores, derived from 2016 Census data. IRSAD scores were grouped into population quintiles and analysed by multinomial regression.

**Results:**

626 participants were included, of whom 155 (25%) were TAB positive. Those with positive TAB were older (76 v 72 years) and a smaller proportion were female (63% v 71%). There was a shift towards a lower SES for patients undergoing TAB, with 161 (26%) in the lowest quintile and 107 (17%) in the highest (p_linear_<0.001). However, SES was not associated with TAB positivity; 34/161 (21%) participants were TAB positive in the lowest quintile compared to 33/107 (31%) in the highest (*p* = 0.19).

**Conclusion:**

SES did not influence incidence of GCA. However, those from lower SES population quintiles were more likely to undergo TAB at a State Pathology service provider. Encouragingly, this suggests there is no issue with access to TAB in SA based on SES.

## Background

Giant Cell Arteritis (GCA) is a systemic granulomatous vasculitis involving medium and large vessels. It is the most common vasculitis affecting the elderly. Age of onset varies depending on disease phenotype, with a mean age of diagnosis 72.1 ± 8.4 years [1]. Historically, GCA was thought to be an inflammatory arteritis involving only branches of the external carotid artery, namely the temporal artery [2, 3], however, it is now recognised as a heterogenous systemic disease which may cause vasculitis of the aorta and any of its major tributaries [4, 5]. Three primary disease subtypes are recognised: classical or ‘pure’ cranial GCA (C-GCA); isolated extracranial large vessel disease without cranial manifestations (LV-GCA); or mixed disease [6]. As such, there are a multitude of possible clinical presentations, ranging from classical symptoms of headache, jaw claudication and visual disturbance, to non-specific constitutional symptoms such as fever, malaise, weight loss, and even limb claudication or stroke [7]. Given lack of clinician awareness regarding the diverse spectrum of disease and limitations in timely access to diagnostic tests, diagnostic delay is a serious issue for patients presenting with GCA, and may result in potentially devastating consequences, including blindness, aortic dissection or stroke [5].

Traditionally, temporal artery biopsy (TAB) was considered the gold standard test for a diagnosis of GCA, however, advanced non-invasive vascular imaging techniques, such as PET, MR-Angiography and doppler ultrasound are increasingly relied upon for diagnosis given the diverse disease spectrum. Timely access to ultrasound by a qualified sonographer or advanced imaging such as PET and MRI is limited, and TAB therefore continues to be the preferred test to confirm a diagnosis of cranial GCA in South Australia (SA) [8]. Accessibility to this highly specialised and invasive test remains a challenge, due to limited availability of specialised surgeons and pathologists, as well as theatre space and time. It is unknown whether other factors, including socioeconomic status (SES), may influence accessibility to TAB in Australia.

A multifactorial aetiology for GCA has been proposed, involving genetic susceptibility, age and environmental triggers [9, 10]. Like other immune-mediated disorders, several environmental exposures have been identified as risk factors for GCA. Although results are conflicting, studies demonstrating seasonal and geographic variation in incidence have implicated a possible infectious trigger [11, 12] whilst correlations with cigarette smoking and pre-existing atherosclerotic disease have also been observed [13, 14]. Other systemic vasculitides have been associated with occupational exposure to inhaled antigens, silica dust and solvents [15, 16]. A number of these environmental factors may be related to SES which may therefore influence both the incidence and outcomes in GCA.

Lower SES has been linked with poorer clinical outcomes in numerous inflammatory rheumatological diseases [17–19]. Several international studies have sought to establish a relationship between SES and GCA. A national Swedish study found that SES defined by occupation, family income and educational level were weakly or inconsistently associated with a propensity to develop GCA [20]. A British study showed that area-level socioeconomic deprivation did not affect rate of TAB positivity, but was associated with increased risk of ischaemic complications, with concern that health seeking behaviours and delay to diagnosis may be responsible for this observation [21]. A second British study proposed that geographical variation in incidence may be related to social class, with higher rates observed in more affluent areas [22], while another found that area-level socioeconomic deprivation in patients with GCA was an independent risk factor for cardiovascular and cerebrovascular disease [23]. Meanwhile, medium-high SES appears to be associated with increased risk of solid organ malignancies in patients with GCA, according to one Israeli study [24].

To our knowledge there have been no previous studies exploring the influence of SES on the likelihood of undergoing a TAB. This is particularly relevant given earlier observations suggesting lower SES is associated with increased risk of ischaemic complications, which may be attributable to diagnostic delay. The aim of this study was to determine whether SES influences access to TAB and rate of biopsy positivity in SA patients with suspected GCA, thereby providing insight as to whether a patient’s home residential address influences access to care.

## Methods

This retrospective cohort study included consecutive patients who underwent TAB examined at SA Pathology between 2017 and 2022. All patients aged 50 years or older, who were resident in South Australia at the time of their biopsy, were included. Patients were excluded if a residential address was not available. SA Pathology processes TAB specimens for both public and private providers. SA Pathology is the largest public pathology service in South Australia. Based on comparative biopsy data available from two private pathology providers between 2017 and 2020 it is estimated that SA Pathology handles approximately 75% of all TAB specimens in South Australia.

All TAB pathology reports during this time period were reviewed and patients with biopsy-proven GCA were identified. Patients were defined as having biopsy-proven GCA if this diagnosis was made by the reviewing pathologist on the diagnostic report. Case note review was undertaken to extract current residential addresses, which were used to classify patients into precise geographical areas known as a ‘Statistical Areas Level 1’ (SA1). SA1s are designated by the Australian Bureau of Statistics’ (ABS) Australian Statistical Geography Standard (ASGS). They are designed using multiple criteria to maximise the geographic detail available for Census of Population and Housing data, and contain a median population of 401 (IQR 315, 501) [25]. For this study, GPS co-ordinates (latitude, longitude) for each address were mapped to their specific SA1 area, using the R library “ASGS” (Interface to the Australian Statistical Geography Standard) [26].

Area-level SES was then determined using the ABS Socio-Economic Indexes for Areas (SEIFA), a suite of indices which provide a summary SES measure for the usual residents within an area, based on 2016 Census data [27]. In this study we applied the Index of Relative Socio-economic Advantage and Disadvantage (IRSAD), which summarises information about the economic and social conditions of people and households within an area, including both relative advantage and disadvantage measures [27]. SA1 area codes for each participant were matched to the corresponding South Australian IRSAD percentile, using information supplied by ABS [27]. For analysis purposes, these were grouped into quintiles (i.e. each representing 20% of the South Australian population ranging from quintile q1 (lowest SES, reflecting lowest 20% of IRSAD scores in SA) to quintile q5 (highest SES, reflecting highest 20% of IRSAD scores in SA).

Statistical analysis was performed using Stata (v16, StataCorp LLC, College Station, TX). Descriptive data for categorical variables are presented as frequencies and percentages, and compared by chi-square tests, while continuous variables are presented as mean with standard deviation (SD), compared by t-tests. All significance tests were two-tailed and values of *p* < 0.05 were considered significant. Statistical analysis of IRSAD quintiles was performed by multinomial logistic regression, with ordinal trends determined using orthogonal polynomial linear contrasts. This study has been approved by the Central Adelaide Local Health Network Human Research Ethics Committee (CALHN HREC) (Reference: 2009145). A waiver of consent was obtained from the CALHN HREC for all participants, as this retrospective population-based study does not contain identifiable data and the benefits from the research were deemed to justify the minimal risks of harm associated with not seeking consent.

## Results

649 biopsy results were available from SA Pathology. 23 cases were excluded: 9 (1.4%) had a non-SA residential address; 7 (1.1%) did not have address details available; and 7 (1.1%) were aged < 50 years. 626 participants were included in the study; of whom 155 (25%) were TAB positive. Participants with positive TAB were older (76 v 72 years) and a smaller proportion were female (63% v 71%), although this observation did not meet statistical significance, as detailed in Table 1.

**Table 1:** Demographics of patients undergoing TAB at SA pathology between 2017–2022

	All	TAB Negative	TAB positive	*p*-val
All Patients n (%)	626	471 (75)	155 (25)	
Females n (%)	431 (69)	334 (71)	97 (63)	0.052
Age mean yrs (sd)	73 (± 10)	72 (± 10)	76 (± 8)	**< 0.001**

TAB: temporal artery biopsy

**Fig. 1:**
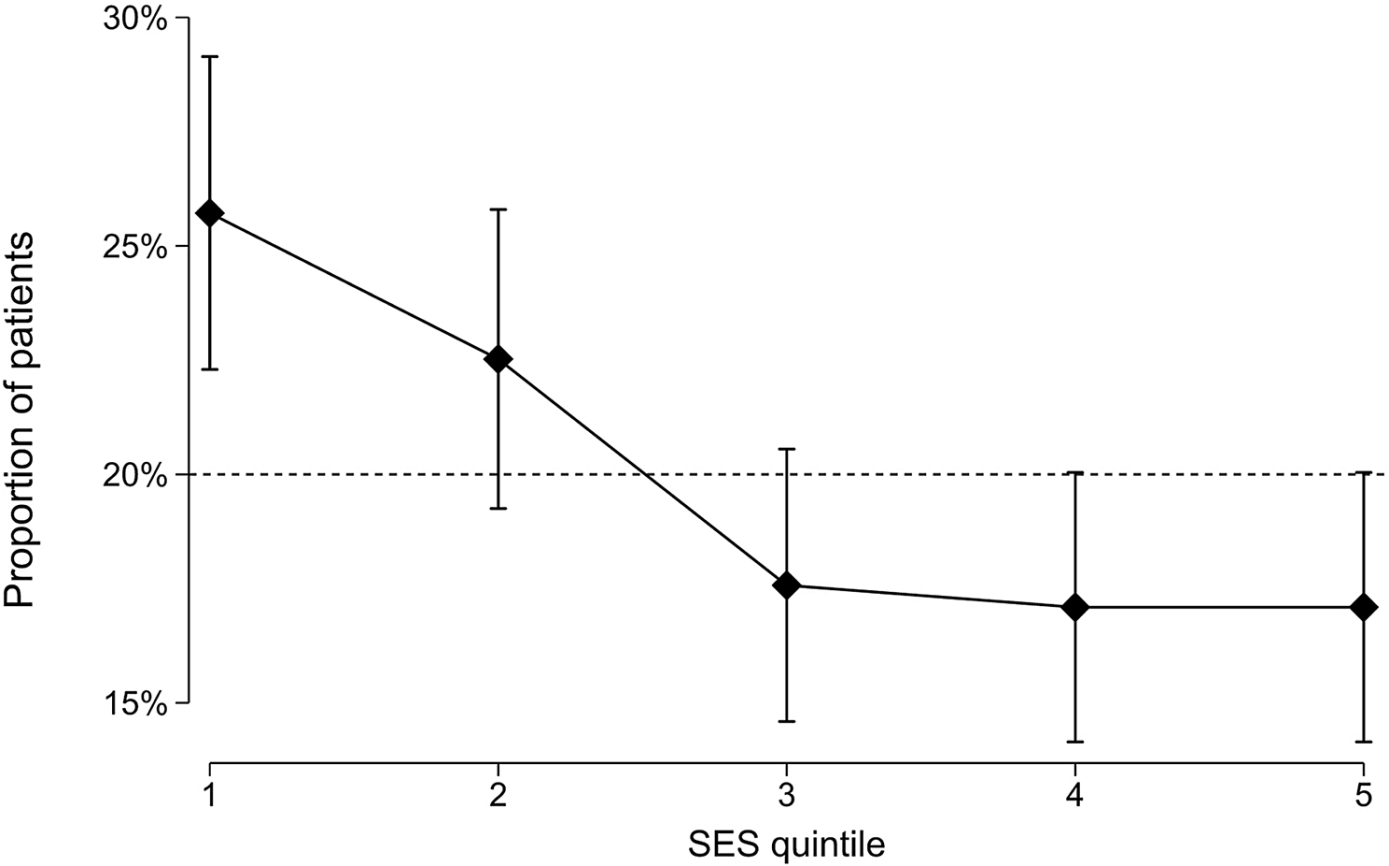
Proportion of patients who underwent a TAB in each IRSAD quintile, relative to the SA population in each quintile (~ 20%). Error bars represent 95% CI. TAB: Temporal Artery Biopsy, IRSAD: Index of Relative Socio-economic Advantage and Disadvantage, SA: South Australian, SES: socioeconomic status, CI: Confidence interval

For patients undergoing TAB, there were 161 participants (26%) in the lowest population quintile and 107 (17%) in the highest. There was an ordinal decrease in the proportion of participants in higher SES quintiles (p_linear_<0.001), indicating a shift towards lower SES for all patients undergoing a TAB with SA Pathology, relative to the SA population (Fig. 1).

Although there was a skew towards lower SES for those who underwent a TAB, compared to the SA population, SES did not appear to be associated with a positive TAB. 34/161 (21%) participants were TAB positive in the lowest population quintile; compared to 33/107 (31%) in the highest (*p* = 0.19). Table 2 summarises the proportion of participants with positive and negative TAB in each IRSAD quintile and demonstrates there is no relationship between TAB result and SES quintile (*p* = 0.29).

**Table 2:** Comparison of proportions for patients with negative and positive TAB in each IRSAD quintile (*p* = 0.29)

IRSAD Quintile (*n*)	TAB	
	Positive *n* (%)	Negative *n* (%)
q1 Lowest (161)	34 (22)	127 (27)
q2 (141)	35 (23)	106 (23)
q3 (110)	31 (20)	79 (17)
q4 (107)	22 (14)	85 (18)
q5 Highest (107)	33 (21)	74 (16)
**Total**	**155**	**471**

IRSAD: Index of Relative Socio-economic Advantage and Disadvantage, TAB: Temporal artery biopsy, Q: quintile

## Discussion

This population-based study found no relationship between SES and incidence of biopsy-positive GCA in South Australia, but did show that people from lower SES population quintiles were more likely to undergo TAB at the state’s largest public pathology service provider. Results are consistent with those of an earlier Swedish study, which demonstrated weak and inconsistent associations between SES and risk of developing GCA [20] and British study which showed area-level socioeconomic deprivation did not affect rate of TAB positivity [21]. Our study is therefore the third of its kind to demonstrate no association between SES and risk of developing GCA. These findings contrast many other immune-mediated diseases, where lower SES and associated occupational exposures have been linked with higher rates of disease, including rheumatoid arthritis (RA), systemic lupus erythematosus (SLE) and ANCA associated vasculitis [15–19].

While our study did not contain individual-level data on patient comorbidities and lifestyle factors, population data suggests that patients in lower SES quintiles have higher rates of smoking and burden of cardiovascular disease (CVD) [28]. In Australia, those in the lowest SES quintile are 3.6 times more likely to smoke, 1.9 times more likely to have diabetes mellitus, and have a 20% increase in age-standardised CVD hospitalisation rate compared to those in the highest SES quintile [29, 30]. Lower SES may therefore be utilised as a surrogate for higher smoking rates and burden of cardiovascular disease. Previous studies have identified smoking and CVD as risk factors for GCA [13, 14, 31], however, our population study size was too small to draw conclusions on association between GCA and smoking status or pre-existing cardiovascular disease.

There are several reasons that lower SES is associated with adverse outcomes in other rheumatic diseases such as RA. These include health literacy, health seeking behaviours, access to health care, and adherence to treatment [17]. Reassuringly, the results of our study suggest that South Australian patients in lowest SES quintiles were not disadvantaged with regards to accessing TAB. This study does not, however, provide information about delay from symptom onset to time of biopsy, and therefore cannot give an indication of diagnostic delay or disease burden at presentation. Mackie et al. demonstrated that socioeconomic deprivation was associated with increased risk of ischaemic complications in GCA, with concern that health seeking behaviours and diagnostic delay may be responsible [21]. Robson et al. demonstrated that lower SES was associated with adverse long-term cardiovascular and cerebrovascular outcomes [23]. Future directions for research in our cohort would be to assess the impact of SES on diagnostic delay, disease outcomes, functional ability, and quality of life.

There are numerous strengths to this study. It is the first of its kind to assess relationship between SES and TAB accessibility and includes a representative sample of the SA population, comprising consecutive TAB specimens from the State Pathology service provider over a 5-year period, derived from both the public and private sector. There are however limitations to this study. It is assumed that SA Pathology handles approximately 75% of TAB specimens in SA, meaning that 25% of biopsy specimens have not been accounted for. We do know that there is the same TAB positivity result in the biopsies performed at both public and private laboratories. The omitted pathology providers are privately operated, processing samples primarily for patients in the private sector. This would likely represent patients in higher SES quintiles and may explain our skewed results suggesting those in lower SES quintiles were more likely to undergo TAB with SA Pathology, relative to the SA population. Another limitation of this study is the use of retrospective data collection and application of population-based area level SES measures. Area level SES measures are prone to ecological fallacy, a statistical bias whereby inferences about an individual are deduced based on the characteristics of a group to which that individual belongs [32]. Patients may have moved address from the time of biopsy to the time that residential addresses data was extracted; although the impact of this is expected to be minimal, as existing Australian research suggests that among individuals who move residential address, the majority move into areas with a similar SES level as the area they moved from [33]. Prospective data collection with application of both personal- and area-level SES measures may improve the validity of our conclusions. Furthermore, this study is limited to a homogenous population of GCA patients who underwent TAB and does not provide information about access to care for the increasing cohort of patients diagnosed with GCA based by advanced non-invasive arterial imaging techniques. The SA GCA registry does not contain population-level data to retrospectively ascertain all cases of GCA diagnosed on imaging; however, this information is being collected prospectively to capture all phenotypes of GCA and better understand healthcare equity across the various subtypes of this heterogenous disease.

This study found that SES did not influence incidence of GCA in South Australia. It did however show that people from lower SES population quintiles were more likely to undergo TAB at a State Pathology service provider. While our data may be skewed somewhat by ascertainment bias, our results are encouraging, and suggest that people from lower SES quintiles are not disadvantaged when it comes to accessing TAB in South Australia.

## Data Availability

The datasets analysed during the current study are not publicly available for confidentiality reasons, but are available from the corresponding author on reasonable request.

## Abbreviations

GCA: Giant Cell Arteritis
TAB: Temporal Artery Biopsy
SES: Socioeconomic Status
SA: South Australia
ABS: Australian Bureau of Statistics
ASGS: Australian Statistical Geography Standard
SEIFA: Socio–Economic Indexes for Areas
IRSAD: Index of Relative Socio–economic Advantage and Disadvantage
CVD: Cardiovascular Disease
RA: Rheumatoid Arthritis
SLE: Systemic Lupus Erythematosus
