# Correction: Influence of socioeconomic status on access to temporal artery biopsy and rates of biopsy positivity in patients with suspected giant cell arteritis

**DOI:** 10.1186/s41927-025-00552-5

**Published:** 2025-08-08

**Authors:** Suellen Anne Lyne, Susan Lester, Oscar Kenneth Russell, Carlee Deanne Ruediger, Kathryn Dyer, Jem Ninan, Ernst Michael Shanahan, Catherine Louise Hill

**Affiliations:** 1 School of Medicine, The University of Adelaide, North Terrace, Adelaide, 5000 South Australia; 2 The Queen Elizabeth Hospital, 28 Woodville Road, Woodville South, Adelaide, 5011 South Australia; 3 Flinders Medical Centre, Flinders Drive, Bedford Park, 5042 South Australia; 4 The Lyell McEwin Hospital, Haydown Road, Elizabeth Vale, 5112 South Australia; 5 The Royal Adelaide Hospital, Port Road, Adelaide, 5000 South Australia

**Correction: BMC Rheumatol (2025) 9:52**

10.1186/s41927-025-00503-0

Following publication of the original article [1], the name of author “Carlee Deanne Ruediger” has been corrected.

The original article [1] has been updated.
